# Sparse Tensor Decomposition for Haplotype Assembly of Diploids and Polyploids

**DOI:** 10.1186/s12864-018-4551-y

**Published:** 2018-03-21

**Authors:** Abolfazl Hashemi, Banghua Zhu, Haris Vikalo

**Affiliations:** 1 Department of ECE, University of Texas at Austin, Austin, Texas, USA; 2 EE Department, Tsinghua University, Beijing, China

**Keywords:** Haplotype assembly, Tensor decomposition, Iterative algorithm

## Abstract

**Background:**

Haplotype assembly is the task of reconstructing haplotypes of an individual from a mixture of sequenced chromosome fragments. Haplotype information enables studies of the effects of genetic variations on an organism’s phenotype. Most of the mathematical formulations of haplotype assembly are known to be NP-hard and haplotype assembly becomes even more challenging as the sequencing technology advances and the length of the paired-end reads and inserts increases. Assembly of haplotypes polyploid organisms is considerably more difficult than in the case of diploids. Hence, scalable and accurate schemes with provable performance are desired for haplotype assembly of both diploid and polyploid organisms.

**Results:**

We propose a framework that formulates haplotype assembly from sequencing data as a sparse tensor decomposition. We cast the problem as that of decomposing a tensor having special structural constraints and missing a large fraction of its entries into a product of two factors, **U** and $\underline {\mathbf {V}}$; tensor $\underline {\mathbf {V}}$ reveals haplotype information while **U** is a sparse matrix encoding the origin of erroneous sequencing reads. An algorithm, AltHap, which reconstructs haplotypes of either diploid or polyploid organisms by iteratively solving this decomposition problem is proposed. The performance and convergence properties of AltHap are theoretically analyzed and, in doing so, guarantees on the achievable minimum error correction scores and correct phasing rate are established. The developed framework is applicable to diploid, biallelic and polyallelic polyploid species. The code for AltHap is freely available from https://github.com/realabolfazl/AltHap.

**Conclusion:**

AltHap was tested in a number of different scenarios and was shown to compare favorably to state-of-the-art methods in applications to haplotype assembly of diploids, and significantly outperforms existing techniques when applied to haplotype assembly of polyploids.

**Electronic supplementary material:**

The online version of this article (10.1186/s12864-018-4551-y) contains supplementary material, which is available to authorized users.

## Background

Fast and accurate DNA sequencing has enabled unprecedented studies of genetic variations and their effect on human health and medical treatments. Complete information about variations in an individual’s genome is given by haplotypes, the ordered lists of single nucleotide polymorphisms (SNPs) on the individual’s chromosomes [1]. Haplotype information is of fundamental importance for a wide range of applications. For instance, when the corresponding genes on a homologous pair of chromosomes contain multiple variants, they could exhibit different gene expression patterns. In humans, this may affect an individual’s susceptibility to diseases and response to therapeutic drugs, and hence suggest directions for medical and pharmaceutical research [2]. Haplotype information also enables whole genome association studies that focus on the so-called tag SNPs [3], representative SNPs in a region of the genome characterized by strong correlation between alleles (i.e., by high linkage disequilibrium). Moreover, haplotype sequences can be used to infer recombination patterns and identify genes under positive selection [4]. In addition to the SNPs and minor structural variations found in a healthy individual’s genome, complex chromosomal aberrations such as translocations and nonreciprocal structural changes – including aneuploidy – are present in cancer cells. Cancer haplotype assembly enables identification of “driver” mutations and thus helps to understanding the mechanisms behind the disease and discovery of its genetic signatures.

Haplotype assembly from short reads obtained by high-through-put DNA sequencing requires partitioning (either directly or indirectly) the reads into *K* clusters (*K*=2 for diploids, *K*=3 for triploids, etc.), each collecting the reads corresponding to one of the chromosomes. If the reads were free of sequencing errors, this task would be straightforward. However, sequencing is erroneous – state-of-the-art platforms have error rates on the order of 10^−3^−10^−2^. This leads to ambiguities regarding the origin of a read and therefore renders haplotype assembly challenging. For this reason, the vast majority of haplotype assembly techniques attempts to remove the aforementioned ambiguities by either discarding or altering sequencing data; this has led to the minimum fragment removal, minimum SNP removal [5], maximum fragments cut [6], and minimum error correction formulations of the assembly problem [7]. Most of the recent haplotype assembly methods (see, e.g., [8–12]) focus on the minimum error correction (MEC) formulation where the goal is to find the smallest number of nucleotides in reads that need to be changed so that any read partitioning ambiguities would be resolved. It has been shown that finding optimal solution to the MEC formulation of the haplotype assembly problem is NP-hard [5, 12, 13]. In [14], the authors used a branch-and-bound scheme to minimize the MEC objective over the space of reads; to reduce the search space, they relied on a bound on the objective obtained by a random partition of the reads. Unfortunately, exponential growth of the complexity of this scheme makes it computationally infeasible even for moderate haplotype lengths. Integer linear programming techniques have been applied to haplotype assembly in [15], but the approach there fails at computationally difficult instances of the problem. More recently, fixed parameter tractable (FPT) algorithms with runtimes exponential in the number of variants per read [16, 17] were proposed; these methods are well-suited for short reads but become infeasible for the long ones. A dynamic programming scheme for haplotype assembly of diploids proposed in [18] is also exponential in the length of the longest read. A probabilistic dynamic programming algorithm that optimizes a likelihood function generalizing the MEC objective is developed in [10]; this method is characterized by high accuracy but is significantly slower than the previous heuristics. Authors in [9, 11] aim to process long reads by developing algorithms for the exact optimization of weighted variants of the MEC score that scale well with read length but are exponential in the sequencing coverage. These methods, along with ProbHap [10], struggle to remain accurate and practically feasible at high coverages (e.g., higher than 12 [10]).

The computational challenges of optimizing MEC score has motivated several polynomial time heuristics. In a pioneering work [19], a greedy algorithm seeking the most likely haplotypes was used to assemble haplotypes of the first complete diploid individual genome obtained via high-throughput sequencing. To compute posterior joint probabilities of consecutive SNPs, Bayesian methods relying on MCMC and Gibbs sampling schemes were proposed in [20] and [21], respectively; unfortunately, slow convergence of Markov chains that these schemes rely on limits their practical feasibility. Following an observation that haplotype assembly can be interpreted as a clustering problem, a max-cut formulation was proposed in [22]; an efficient algorithm (HapCUT, recently upgraded to HapCUT2 [23]) that solves it and significantly outperforms the method in [19] was developed and has widely been used. A flow-graph based approach in [24], HapCompass, re-examined fragment removal strategy and demonstrated superior performance over HapCUT. Other recent diploid haplotype assembly methods include a greedy max-cut approach in [25], convex optimization program for minimizing the MEC score in [26], a communication-theoretic interpretation of the problem solved via belief propagation (BP) in [27], and methods that use external reference panels such as 1000 Genomes to improve accuracy of haplotype assembly in [28, 29]. Note that deep sequencing coverage provided by state-of-the-art high-throughput sequencing platforms and the emergence of very long insert sizes in recent technologies (e.g., fosmid [25]) may enable assembly of extremely long haplotype blocks but also impose significant computational burden on the methods above.

Increased affordability, capability to provide deep coverage, and longer sequencing read lengths also enabled studies of genetic variations of polyploid organisms. However, haplotype assembly for polyploid genomes is considerably more challenging than that for diploids; to illustrate this, note that for a polyploid genome with *k* haplotype sequences of length *m*, under the all-heterozygous assumption there are (*k*−1)^*m*^ different genotypes and at least 2^(*m*−1)^(*k*−1)^*m*^ different haplotype phasings. In part for this reason relatively fewer methods for solving the haplotype assembly problems in polyploids have been developed. In fact, with the exception of HapCompass [24], SDhaP [26] and BP [27], the above listed methods are restricted to diploid genomes. Other techniques capable of reconstructing haplotypes for both diploid and polyploid genomes include HapTree [30], a Bayesian method to find the maximum likelihood haplotype shown to be superior to HapCompass and SDhaP (see, e.g., [31] for a detailed comparison), H-PoP [8], the state-of-the-art dynamic programming method that significantly outperforms the schemes developed in [24, 26, 30] in terms of accuracy, memory consumption, and speed, and the recently proposed matrix factorization schemes in [32, 33].

In this paper, we propose a unified framework for haplotype assembly of diploid and polyploid genomes based on sparse tensor decomposition; the framework essentially solves a relaxed version of the NP-hard MEC formulation of the haplotype assembly problem. In particular, read fragments are organized in a sparse binary tensor which can be thought of as being obtained by multiplying a matrix that contains information about the origin of erroneous sequencing reads and a tensor that contains haplotype information of an organism. The problem then is recast as that of decomposing a tensor having special structural constraints and missing a large fraction of its entries. Based on a modified gradient descent method and after unfolding the observed and haplotype information bearing tensors, an iterative procedure for finding the decomposition is proposed. The algorithm exploits underlying structural properties of the factors to perform decomposition at a low computational cost. In addition, we analyze the performance and convergence properties of the proposed algorithm and determine bounds on the minimum error correction (MEC) scores and correct phasing rate (CPR) – also referred to as reconstruction rate – that the algorithm achieves for a given sequencing coverage and data error rate. To the best of our knowledge, this is the first polynomial time approximation algorithm for haplotype assembly of diploids and polyploids having explicit theoretical guarantees for its achievable MEC score and CPR. The proposed algorithm, referred to as AltHap, is tested in applications to haplotype assembly for both diploid and polyploid genomes (synthetic and real data) and compared with several state-of-the-art methods. Our extensive experiments reveal that AltHap outperforms the competing techniques in terms of accuracy, running time, or both. It should be noted that while state-of-the-art haplotype assembly methods for polyploids assume haplotypes may only have biallelic sites, AltHap is capable of reconstructing polyallelic haplotypes which are common in many plants and some animals, are of particular importance for applications such as crop cultivation [34], and may help in reconstruction of viral quasispecies [35]. Moreover, while many state-of-the-art haplotype assembly methods are computationally intensive (e.g., [10, 15]), our extensive numerical experiments demonstrate efficacy of AltHap in a variety of practical settings.

## Methods

### Problem formulation

We briefly summarize notation used in the paper. Bold capital letters refer to matrices and bold lowercase letters represent vectors. Tensors are denoted by underlined bold capital letters, e.g., $\underline {\mathbf {M}}$. **M**_::1_ and $\overline {\mathbf {M}}$ denote the frontal slice and the mode-1 unfolding of a third-order tensor $\underline {\mathbf {M}}$, respectively. For a positive integer *n*, [*n*] denotes the set {1…,*n*}. The condition number of rank-*k* matrix **M** is defined as *κ*=*σ*_1_/*σ*_*k*_ where *σ*_1_≥⋯≥*σ*_*k*_>0 are singular values of **M**. SVD_*k*_(**M**) denotes the rank *k* approximation (compact SVD) of **M** computed by power iteration method [36, 37].

Let ${\mathcal {H}}=\{\mathbf {h}_{1},\dots,\mathbf {h}_{k}\}$ denote the set of haplotype sequences of a *k*-ploid organism, and let **R** be an *n*×*m* SNP fragment matrix where *n* denotes the number of sequencing reads and *m* is the length of haplotype sequences. **R** is an incomplete matrix that can be thought of as being obtained by sampling, with errors, matrix **M** that consists of *n* rows; each row of **M** is a sequence randomly selected from among *k* haplotype sequences. Since each SNP is one of four possible nucleotides, we use the alphabet ${\mathcal {A}}=\{(1,0,0,0),(0,1,0,0),(0,0,1,0),(0,0,0,1)\}$ to describe the information in the haplotype sequences; the mapping between nucleotides and alphabet components follows arbitrary convention. The reads can now be organized into an *n*×*m*×4 SNP fragment tensor which we denote by $\underline {\mathbf {R}}$. The (*i*,*j*,:) fiber of $\underline {\mathbf {R}}$, i.e., a one-dimensional slice obtained by fixing the first and second indices of the tensor, represents the value of the *j*^*th*^ SNP in the *i*^*th*^ read. Let *Ω* denote the set of informative fibers of $\underline {\mathbf {R}}$, i.e., the set of (*i*,*j*,:) such that the *i*^*th*^ read covers the *j*^*th*^ SNP. Define an operator ${\mathcal {P}}_{\Omega }(.)$ as

1 $$ \left[{\mathcal{P}}_{\Omega}(\underline{\mathbf{R}})\right]_{ij:}=\left\{ \begin{array}{ll} \mathbf{R}_{ij:}& (i, j,:) \in \Omega\\ \mathbf{0},& \text{otherwise.} \end{array}\right.  $$

**Fig. 1:**
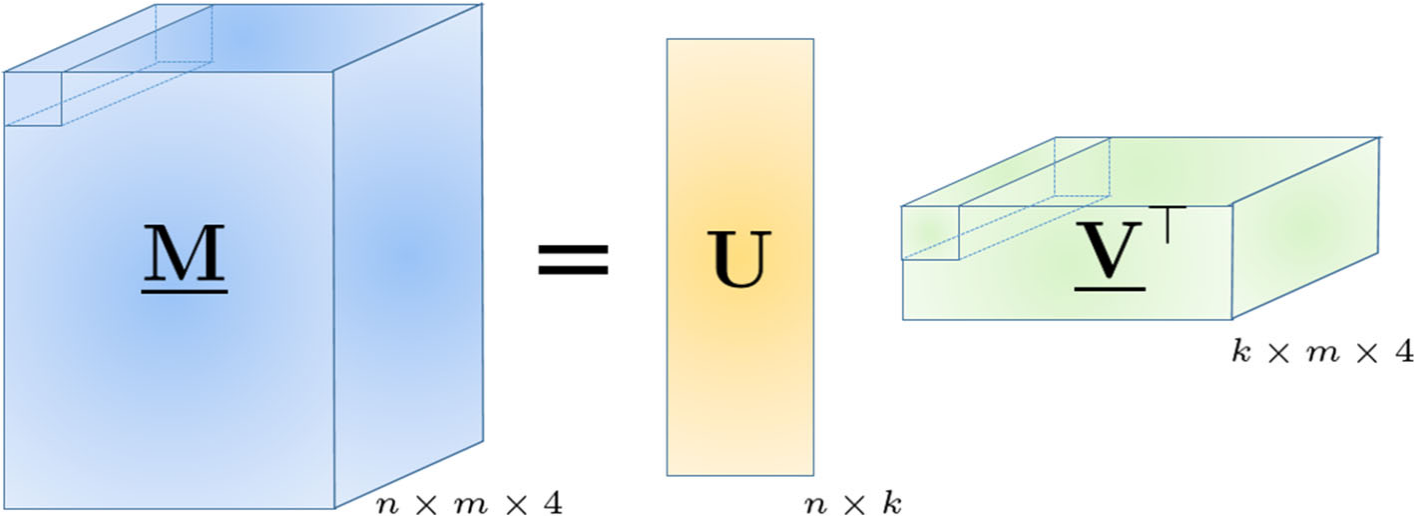
Representing haplotype sequences and sequencing reads using tensors. Tensor $\underline {\mathbf {V}} \in {\mathcal {A}}^{m \times k}$ contains haplotype information while matrix **U**∈{0,1}^*n*×*k*^ assigns each of the *n* horizontal slices of $\underline {\mathbf {M}}$ to one of the *k* haplotype sequences, i.e., the *i*^*th*^ row of **U** is an indicator of the origin of the *i*^*th*^ read

${\mathcal {P}}_{\Omega }(\underline {\mathbf {R}})$ is a tensor obtained by sampling, with errors, tensor $\underline {\mathbf {M}} \in {\mathcal {A}}^{n \times m}$ having *n* copies of *k* encoded haplotype sequences as its horizontal slices. More specifically, we can write $\underline {\mathbf {M}}=\mathbf {U}\underline {\mathbf {V}}^{\top }$, where $\underline {\mathbf {V}} \in {\mathcal {A}}^{m \times k}$ contains haplotype information, i.e., the *j*^*th*^ vertical slice of $\underline {\mathbf {V}}$, **V**_:*j*:_, is the encoded sequence of the *j*^*th*^ haplotype, and **U**∈{0,1}^*n*×*k*^ is a matrix that assigns each of *n* horizontal slices of $\underline {\mathbf {M}}$ to one of *k* haplotype sequences, i.e., the *i*^*th*^ row of **U**, **u**_*i*_, is an indicator of the origin of the *i*^*th*^ read. Let *Φ*={**e**_1_,…,**e**_*k*_}, where $\mathbf {e}_{l} \in \mathbb {R}^{k}$ is the *l*^*th*^ standard basis vector having 1 in the *l*^*th*^ position and 0 elsewhere. The rows of **U** are standard unit basis vectors in $\mathbb {R}^{k}$, i.e., **u**_*i*_∈*Φ*, ∀*i*∈[*n*]. This representation is illustrated in Fig. 1 where the (1,1,:) fiber of $\underline {\mathbf {V}}$ specified with dashed lines is mapped to the (1,1,:) fiber of $\underline {\mathbf {M}}$ which in turn implies that in the example described in Fig. 1 we have **u**_1_=**e**_1_.

DNA sequencing is erroneous and hence we assume a model where the informative fibers in $\underline {\mathbf {R}}$ are perturbed versions of the corresponding fibers in $\underline {\mathbf {M}}$ with data error rate *p*_*e*_, i.e., if the (*i*,*j*,:)∈*Ω* fiber in $\underline {\mathbf {M}}$ takes value $\mathbf {e}_{l} \in {\mathcal {A}}$, **R**_*ij*:_ with probability 1−*p*_*e*_ equals **e**_*l*_ and with probability *p*_*e*_ takes one of the other three possibilities. Thus, the observed SNP fragment tensor can be modeled as $\underline {\mathbf {R}}={\mathcal {P}}_{\Omega }(\underline {\mathbf {M}}+\underline {\mathbf {N}})$ where $\underline {\mathbf {N}}$ is an additive noise tensor defined as

2 $$ \mathbf{N}_{ij:}=\left\{ \begin{array}{cc} \mathbf{0}, &\text{ w.p}\quad 1-p_{e} \\ {\mathcal{U}}({\mathcal{A}}\backslash\{\mathbf{M}_{ij:}\})-\mathbf{M}_{ij:},&\text{ w.p}\quad p_{e}, \end{array}\right.  $$

where the notation ${\mathcal {U}}({\mathcal {A}}\backslash \{\mathbf {M}_{ij:}\})$ denotes uniform selection of a vector from ${\mathcal {A}}\backslash \{\mathbf {M}_{ij:}\}$. The goal of haplotype assembly can now be formulated as follows: *Given the SNP fragment tensor*$\underline {\mathbf {R}}$, *find the tensor of haplotype sequences*$\underline {\mathbf {V}}$*that minimizes the MEC score*.

Next, we formalize the MEC score as well as the correct phasing rate, also known as reconstruction rate, the two metrics that are used to characterize performance of haplotype assembly schemes (see, e.g., [15, 18, 38, 39]). For two alleles **a**_1_, $\mathbf {a}_{2} \in {\mathcal {A}}\cup \{\mathbf {0}\}$, we define a dissimilarity function *d*(**a**_1_,**a**_2_) as

3 $$ d(\mathbf{a}_{1},\mathbf{a}_{2})=\left\{ \begin{matrix} 1,& \text{if } \mathbf{a}_{1},\mathbf{a}_{2}\neq \mathbf{0} \text{ and } \mathbf{a}_{1}\neq\mathbf{a}_{2}\\ 0,& \text{otherwise.} \end{matrix}\right.  $$

The MEC score is the smallest number of fibers in $\underline {\mathbf {R}}$ that need to be altered so that the resulting modified data is consistent with the reconstructed haplotype $\underline {\mathbf {V}}$, i.e.,

4 $$ \text{MEC}=\sum_{i=1}^{n} \min_{p=1,\dots,k} \sum_{j=1}^{m} d(\mathbf{R}_{ij:},\mathbf{V}_{jp:}).  $$

The correct phasing rate (CPR), also referred to as the reconstruction rate, can conveniently be written using the dissimilarity function *d*(.,.). Let $\underline {\mathbf {V}}^{t}$ denote the tensor of true haplotype sequences. Then

5 $$ \text{CPR}=1-\frac{1}{mk}\left(\min_{\mathcal{M}} \sum_{i=1}^{m}\sum_{j=1}^{k} d\left({\mathcal{M}}(\underline{\mathbf{V}})_{ij:},\mathbf{V}_{ij:}^{t}\right)\right),  $$

where $\mathcal {M}$ is a one-to-one mapping from lateral slices of $\underline {\mathbf {V}}$ to those of $\underline {\mathbf {V}}^{t}$, i.e., a one-to-one mapping from the set of reconstructed haplotypes to the set of true haplotypes.

We now describe our proposed relaxation of the MEC formulation of the haplotype assembly problem. Let *p*_*i*_∈[*k*], ∀*i*∈[*n*] be defined as $p_{i}= \arg \min _{p} \sum _{j=1}^{m} d\left (\mathbf {R}_{ij:},{\mathbf {V}}_{jp:}\right)$. Notice that for any *j* such that *d*(**R**_*ij*:_,**V**_*jp*:_)=1, $\|\mathbf {R}_{ij:}-{\mathbf {V}}_{jp:}\|_{2}^{2}=2$. Therefore, by denoting $\Omega =\cup _{i=1}^{n}\Omega _{i}$ where *Ω*_*i*_ the set of informative fibers for the *i*^*th*^ read we obtain

6 $$ \begin{aligned} p_{i}&= \arg\min_{p} \sum_{j=1}^{m} d\left(\mathbf{R}_{ij:},{\mathbf{V}}_{jp:}\right)\\ & = \frac{1}{2}\arg\min_{p}\sum_{j=1}^{m}\|\mathbf{R}_{ij:}-{\mathcal{P}}_{\Omega_{i}}\left({\mathbf{V}}_{jp:}\right)\|_{2}^{2} \\ &\stackrel{(a)}{=} \frac{1}{2}\arg\min_{p}\|\mathbf{R}_{i::}-{\mathcal{P}}_{\Omega_{i}}\left({\mathbf{V}}_{:p:}\right)\|_{F}^{2}\\ &\stackrel{(b)}{=} \frac{1}{2}\arg\min_{p}\|\text{vec}(\mathbf{R}_{i::})-\text{vec}\left({\mathcal{P}}_{\Omega_{i}}\left({\mathbf{V}}_{:p:}\right)\right)\|_{2}^{2} \end{aligned}  $$

where (*a*) follows from the definition of the Frobenius norm and vec(.) in (*b*) denotes the vectorization of its argument. Let **e**_*p*_ be the *p*^*th*^ standard unit vector ∀*p*∈[*k*]. It is straightforward to observe that the last equality in (6) can equivalently be written as

$$p_{i}=\frac{1}{2}\arg\min_{p}\|\text{vec}(\mathbf{R}_{i::})-{\mathcal{P}}_{\Omega_{i}}\left(\overline{\mathbf{V}}\mathbf{e}_{p}\right)^{\top}\|_{2}^{2} $$

where $\overline {\mathbf {V}}$ is the mode-1 unfolding of the tensor $\underline {\mathbf {V}}$. Hence,

$$\text{MEC}=\frac{1}{2}\sum_{i=1}^{n} \|\text{vec}(\mathbf{R}_{i::})-{\mathcal{P}}_{\Omega_{i}}\left(\overline{\mathbf{V}}\mathbf{e}_{p}\right)^{\top}\|_{2}^{2}. $$

Let **U**∈{0,1}^*n*×*k*^ be the matrix such that for its *i*^*th*^ row it holds that $\mathbf {u}_{i} = \mathbf {e}_{p_{i}}\phantom {\dot {i}\!}$. In addition, notice that vec(**R**_*i*::_) is the *i*^*th*^ row of $\overline {\mathbf {R}}$. Therefore, from the definition of the Frobenius norm and the fact that ${\mathcal {P}}_{\Omega }(\overline {\mathbf {R}})=\overline {\mathbf {R}}$ we obtain

7 $$ \text{MEC}=\min_{\mathbf{U},\overline{\mathbf{V}}}\frac{1}{2}\left\|{\mathcal{P}}_{\Omega}\left(\overline{\mathbf{R}}-\mathbf{U}\overline{\mathbf{V}}^{\top}\right)\right\|_{F}^{2}.  $$

The optimization problem in (7) is NP-hard since the entries of $\overline {\mathbf {V}}$ are binary and the objective function is non-convex. Relaxing the binary constraint to $\overline {\mathbf {V}}_{i,j} \in {\mathcal {C}}$, ∀*i*∈[4*m*],∀*j*∈[*k*], where ${\mathcal {C}}=[0,1]$, results in the following relaxation of the MEC formulation,

8 $$ \begin{aligned} & \underset{\mathbf{U},\overline{\mathbf{V}}}{\text{min}} \quad \frac{1}{2}\left\|{\mathcal{P}}_{\Omega}\left(\overline{\mathbf{R}}-\mathbf{U}\overline{\mathbf{V}}^{\top}\right)\right\|^{2}_{F}\\ & \text{s.t.}\hspace{0.5cm}\overline{\mathbf{V}}_{i,j} \in {\mathcal{C}}, \forall i \in [4m], \forall j \in [k]\\ &\hspace{0.85cm} \mathbf{u}_{i} \in \Phi, \forall i \in [n]. \end{aligned}  $$

**Fig. 2:**
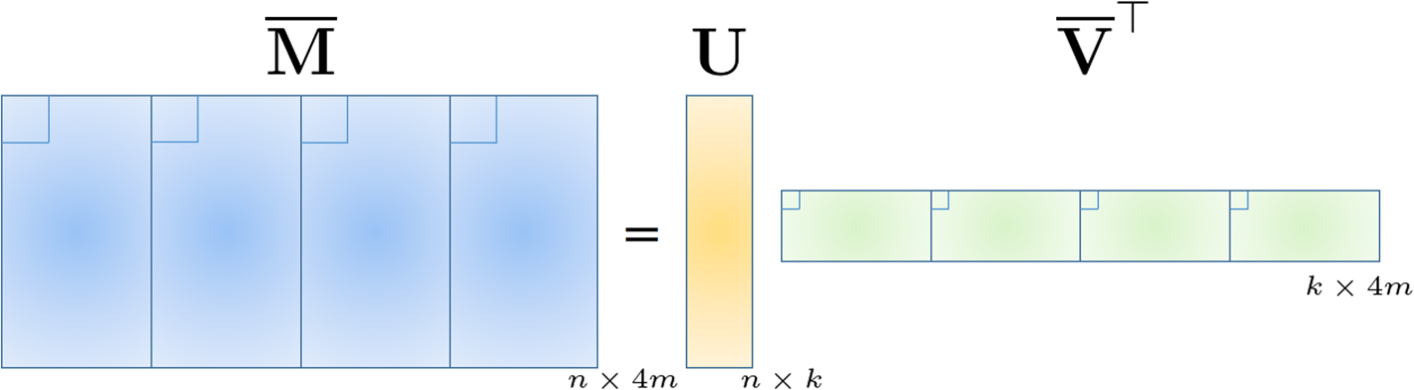
Representing haplotype sequences and sequencing reads using unfolded tensors. Matrix $\overline {\mathbf {V}} \in \{0,1\}^{4m \times k}$ contains haplotype information while matrix **U**∈{0,1}^*n*×*k*^ assigns each of the *n* rows of $\overline {\mathbf {M}}$ to one of the *k* haplotype sequences, i.e., the *i*^*th*^ row of **U** is an indicator of the origin of the *i*^*th*^ read

The new formulation can be summarized as follows. We start by finding the so-called mode-1 unfolding of tensors $\underline {\mathbf {M}}$ and $\underline {\mathbf {V}}$ and denote the decomposition $\overline {\mathbf {M}}=\mathbf {U}\overline {\mathbf {V}}^{\top }$, as illustrated in Fig. 2. As implied by the figure, after unfolding, the entries of the (1,1,:) fiber are mapped to four blocks of $\overline {\mathbf {M}}$ and $\overline {\mathbf {V}}$ that correspond to the frontal slices of tensors $\underline {\mathbf {M}}$ and $\underline {\mathbf {V}}$, respectively. Then, to determine the haplotype sequence that minimizes the MEC score, one needs to solve (8) and find the optimal tensor decomposition.

### The AltHap algorithm

Although the objective function in (8), i.e.,

$${{f(\mathbf{U},\overline{\mathbf{V}})=\frac{1}{2}\|{{\mathcal{P}}_{\Omega}}\left(\overline{\mathbf{R}}-\mathbf{U}\overline{\mathbf{V}}^{\top}\right)\|^{2}_{F}}} $$

is convex in each of the factors when the other factor is fixed, $f(\mathbf {U},\overline {\mathbf {V}})$ is generally nonconvex. To facilitate computationally efficient search for the solution of (8), we rely on a modified gradient search algorithm which exploits the special structures of **U** and $\overline {\mathbf {V}}$ and iteratively updates the estimates $(\mathbf {U}_{t},\overline {\mathbf {V}}_{t})$ starting from some initial point $(\mathbf {U}_{0} \overline {\mathbf {V}}_{0})$. More specifically, given the current estimates $(\mathbf {U}_{t},\overline {\mathbf {V}}_{t})$, the update rules are

9 $$ \mathbf{U}_{t+1}=\arg\min_{\mathbf{u}_{i} \in \Phi}{\sum_{(i,j)\in \Omega}\left\|{\mathcal{P}}_{\Omega}\left(\overline{\mathbf{R}}-\mathbf{U}_{t}\overline{\mathbf{V}}_{t}^{\top}\right)\right\|_{F}^{2}}  $$

10 $$ \overline{\mathbf{V}}_{t+1}={{\Pi_{\mathcal{C}}}}\left(\overline{\mathbf{V}}_{t}-\alpha{{\nabla f}}(\overline{\mathbf{V}}_{t})\right),  $$

where ${{\nabla f}} \left (\overline {\mathbf {V}}_{t}\right) = -\left ({\mathcal {P}}_{\Omega }\left (\overline {\mathbf {R}}-\mathbf {U}_{t+1}\overline {\mathbf {V}}_{t}^{\top }\right)\right)^{\top }\mathbf {U}_{t+1}$ denotes the partial derivative of $f\left (\mathbf {U},\overline {\mathbf {V}}\right)$ evaluated at $\left (\mathbf {U}_{t+1},\overline {\mathbf {V}}_{t}\right)$, *α* is a judiciously chosen step size, and ${{\Pi _{\mathcal {C}}}}$ denotes the projection operator onto ${\mathcal {C}}$. Notice that the optimization in (9) is done by exhaustively searching over *k* vectors in *Φ*. Since the number of haplotypes *k* is relatively small, the complexity of the exhaustive search (9) is low. The proposed scheme is formalized as Algorithm 1.



Note that AltHap differs from a previously proposed SCGD algorithm in [32] as follows: (i) AltHap’s novel representation of haplotypes and sequencing reads using binary tensors provides a unified framework for haplotype assembly of diploids as well as biallelic and polyallelic polyploids. The method in [32] is not capable of performing haplotype assembly of polyallelic polyploid genomes. (ii) Unlike [32], AltHap exploits the fact that $\underline {\mathbf {V}}$ is composed of binary entries by imposing the constraint $\overline {\mathbf {V}}_{i,j} \in {\mathcal {C}}$ in the MEC relaxation in (8). As our results in Section 5 demonstrate, this leads to significant performance improvements of AltHap over SCGD in a variety of settings. (iii) Lastly, in Section 4 we provide analysis of the global convergence of AltHap and derive explicit analytical bounds on its achievable performance. Such performance guarantees do not exist for the method in [32].

### Convergence analysis of AltHap

In this section, we analyze the convergence properties of AltHap and provide performance guarantees in different scenarios.

In the Additional file 1 we show that, a judicious choice of the step size *α* according to

11 $$ \alpha=\frac{C\|{{\nabla f}}\left(\overline{\mathbf{V}}_{t}\right)\|_{F}^{2}}{\left\|{\mathcal{P}}_{\Omega}\left(\mathbf{U}_{t+1}{{\nabla f}}\left(\overline{\mathbf{V}}_{t}\right)^{\top}\right)\right\|_{F}^{2}},  $$

where *C*∈(0,2) is a constant, guarantees that the value of the objective function in (8) decreases as one alternates between (9) and (10), which in turn implies that AltHap converges. The key observation that leads to this result is that $f(\mathbf {U},\overline {\mathbf {V}})$ is a convex function in each of the factor matrices and that ${\mathcal {C}}=[0,1]$ is a convex set; hence the projection ${{\Pi _{\mathcal {C}}}}$ in (10) leads to a reduction of $f\left (\mathbf {U}_{t},\overline {\mathbf {V}}_{t}\right)$ in each iteration *t*.

It is important however to determine the conditions under which the stationary point of AltHap coincides with the global optima of (8). To this end, we first provide the definition of incoherence of matrices [40].

#### **Definition 1**

*A rank-k matrix*$\mathbf {M} \in \mathbb {R}^{n \times m}$*with singular value decomposition*$\mathbf {M}=\hat {\mathbf {U}}\mathbf {\Sigma }\hat {\mathbf {V}}^{\top }$*is incoherent with parameter*$1\leq \mu \leq \frac {\max \{n,m\}}{k}$*if for every* 1≤*i*≤*n*, 1≤*j*≤*m*

12 $$ \sum_{l=1}^{k}{\hat{\mathbf{U}}_{il}^{2}\leq \frac{\mu k}{n}},\quad \sum_{l=1}^{k}{\hat{\mathbf{V}}_{jl}^{2}\leq \frac{\mu k}{m}}.  $$

Let each fiber in **M***T* be observed uniformly with probably *p*. Let *C*_snp_=△*mp* denote the expected number of SNPs covered by each read, and *C*_seq_=△*np* denote the expected coverage for each of the haplotype sequences. Theorem 1 built upon the results of [41–43] states that with an adequate number of covered SNPs, the solution found by AltHap reconstructs $\overline {\mathbf {M}}$ up to an error term that stems from the existence of errors in sequencing reads.

#### **Theorem 1**

Assume $\overline {\mathbf {M}}$ is *μ*-incoherent. Suppose the condition number of $\overline {\mathbf {M}}$ is *κ*. Then there exist numerical constants *C*_0_,*C*_1_>0 such that if *Ω* is uniformly generated at random and

13 $$ C_{\text{snp}} > \max\left\{ C_{0} \sqrt[3]{\mu^{4}k^{14}\kappa^{12} C_{\text{seq}}},\frac{p_{e} k^{2} \kappa^{6}}{2C_{1}}\right\}  $$

with probability at least $1-\frac {1}{m^{3}}$, the solution $(\mathbf {U}^{*}, \overline {\mathbf {V}}^{*})$ found by AltHap satisfies

14 $$ \left\|\overline{\mathbf{M}}-\mathbf{U}^{*}{\overline{\mathbf{V}}^{*}}^{\top}\right\|_{F}^{2} \leq \frac{C_{1} \kappa^{4} p_{e} k m}{2C_{\text{snp}}}.  $$

The proof of Theorem 1 relies on a coupled perturbation analysis to establish a certain type of local convexity of the objective function around the global optima. Thus, under (13) there is no other stationary point around the global optima and hence, starting from a good initial point, AltHap converges globally. We employ the initialization procedure suggested by [42] – summarized in the initialization step of Algorithm 1 – which is based on a low cost singular value decomposition of $\overline {\mathbf {R}}$ using power method [36, 37] and with high probability lies in the described convexity region of $f(\mathbf {U},\overline {\mathbf {V}})$.

#### **Remark 1**

Under the assumption of 1, the Condition $C_{\text {snp}} > C_{0} \sqrt [3]{\mu ^{4}k^{14}\kappa ^{12} C_{\text {seq}}}$ specifies a lower bound on the expected number of covered SNPs, *C*_snp_, that is required for the exact recovery of $\overline {\mathbf {M}}$ in the idealistic error-free scenario, i.e., for *p*_*e*_=0. With higher sequencing coverage, more SNPs are covered by the reads and hence *C*_snp_ required for accurate haplotype assembly scales with *C*_seq_ along with other parameters. Moreover, the term $\frac {C_{1} \kappa ^{4} p_{e} k m}{2C_{\text {snp}}}$ on the right hand side of (14) is the bound on the error of the solution generated by AltHap which increases with the sequencing error rate *p*_*e*_ and ploidy *k*, and decreases with *C*_snp_ and the number of reads *n*, as expected.

#### **Remark 2**

If $\overline {\mathbf {M}}$ is well-conditioned, i.e., $\overline {\mathbf {M}}$ is characterized by a small incoherence parameter *μ* and a small condition number *κ*, the recovery becomes easier; this is reflected in less strict sufficient condition (13) and improved achievable performance (14). In fact, as we verified in our simulation studies, by using the proposed framework for haplotype assembly, the parameters *μ* and *κ* associated with $\overline {\mathbf {M}}$ are close to 1 (the ideal case). Theorem 2 provides theoretical bounds on the expected MEC scores and CPR achieved by AltHap. (See Additional file 1 for the proof).

#### **Theorem 2**

Under the conditions of Theorem 1, with probability at least $1-\frac {1}{m^{3}}$ it holds that

15 $$ \mathbb{E}\{\text{MEC}\} \leq 2 p_{e} \left(C_{\text{seq}}m+\kappa^{4}C_{1}k\right).  $$

Moreover, if the reads sample haplotype sequences uniformly, with probability at least $1-\frac {1}{m^{3}}$ it holds that

16 $$ \mathbb{E}\{\text{CPR}\} \geq 1-\frac{C_{1} \kappa^{4} p_{e} k}{nC_{\text{snp}}}.  $$

#### **Remark 3**

The bound established in (15) suggests that the expected MEC increases with the length of the haplotype sequences, sequencing error, number of haplotype sequences, and sequencing coverage. A higher sequencing coverage results in a larger fragment data which in turn leads to higher MEC scores.

#### **Remark 4**

As intuitively expected, the bound (16) suggests that AltHap’s achievable expected CPR improves with the number of sequencing reads and the SNP coverage; on the other hand, the CPR deteriorates at higher data error rates. Finally, assuming the same sequencing parameters, (16) implies that reconstruction of polyploid haplotypes is more challenging than that of diploids.

## Results and discussion

We evaluated the performance of the proposed method on both experimental and simulated data, as described next. AltHap was implemented in Python and MATLAB, and the simulations were conducted on a single core Intel Xeon E5-2690 v3 (Haswell) with 2.6 GHz and 64 GB DDR4-2133 RAM. The benchmarking algorithms include Belief Propagation (BP) [27], a communication-inspired method capable of performing haplotype assembly of diploid and biallelic polyploid species, HapTree [30], integer linear programming (ILP) technique [15], SCGD [32], and H-PoP [8], the state-of-the-art dynamic programming algorithm for haplotype assembly of diploid and biallelic polyploid species shown to be superior to HapTree [30], HapCompass [24], and SDhaP [26] in terms of both accuracy and speed [8, 31]. Following the prior works on haplotype assembly (see, e.g., [15, 18, 38, 39]) we use MEC score and CPR to assess the quality of the reconstructed haplotypes. For clarity, in the tables we report the CPR percentage, i.e., CPR × 100.

### Experimental data

We first tested performance of AltHap in an application to haplotype reconstruction of a data set from the 1000 Genomes Project – in particular, the sample NA12878 sequenced at high coverage using the 454 sequencing platform. In this work, we take the trio-phased variant calls from the GATK resource bundle [44] as the true haplotype sequences. We compare the MEC score, CPR, and running time achieved by AltHap to those of H-PoP, BP, HapTree, SCGD and ILP. All the algorithms used in the benchmarking study were executed with their default settings. The results are given in Table 1. As seen there, among the considered algorithms AltHap achieves the highest CPR for majority of the chromosomes (nine), followed by H-PoP and ILP (five each). As expected, ILP achieves the lowest MEC scores among all the methods but this comes at a computational cost much higher than that of AltHap. Notice that lower MEC score does not necessarily imply better CPR. MEC is the error evaluated on observed SNPs positions, i.e., the training data points, while CPR is related to the generalization error that is calculated on unobserved SNPs positions, i.e., the test data points. Since the sequencing reads are erroneous, an algorithm might over-fit while trying to minimize the MEC score.

**Table 1:** Performance comparison of AltHap, H-PoP, BP, HapTree, SCGD, and ILP applied to haplotype reconstruction of the CEU NA12878 data set in the 1000 Genomes Project

	AltHap			H-PoP			BP		
Chromosome	CPR	MEC	t(sec)	CPR	MEC	t(sec)	CPR	MEC	t(sec)
1	97.4	2011	11.26	95.7	2264	5.22	99.1	2321	8.17
2	95.3	2562	12.22	95.6	2971	5.65	89.5	2897	9.83
3	93.3	2084	10.38	91.2	2312	6.99	74.3	2367	8.30
4	96.9	2368	12.16	97.0	2648	5.24	74.8	2613	6.76
5	97.2	1924	9.96	96.6	2103	4.67	88.2	2185	4.76
6	94.9	3687	14.17	95.2	3343	4.93	88.7	3588	6.94
7	97.0	1846	11.19	92.4	1986	4.24	81.1	2073	7.88
8	96.2	1634	9.63	94.7	1848	4.14	88.5	1857	8.01
9	97.1	1272	6.42	91.0	1462	3.36	89.8	1491	6.13
10	96.8	1584	7.97	94.5	1683	3.67	90.8	1839	7.18
11	93.3	1394	7.45	91.5	1553	3.71	75.6	1586	6.69
12	92.1	1423	7.12	90.3	1570	3.46	74.4	1589	6.48
13	97.0	1269	4.42	94.1	1440	2.89	89.1	1409	5.38
14	90.3	857	9.53	97.1	974	2.54	70.0	995	4.53
15	97.2	941	9.42	97.4	1039	2.40	74.6	1063	3.92
16	96.7	1198	5.40	93.5	1192	2.47	79.7	1269	4.42
17	97.5	1146	4.58	91.1	1244	1.98	92.4	1234	3.15
18	91.0	860	4.54	97.6	893	2.51	82.0	942	3.79
19	97.6	618	3.32	97.8	695	1.82	98.0	1060	2.47
20	97.3	703	3.53	95.0	719	2.00	97.1	796	2.74
21	97.4	470	2.51	97.0	512	1.70	97.5	532	1.86
22	97.3	367	1.98	98.3	427	1.44	90.7	438	1.72
Mean	95.8	1464	7.69	94.8	1585	3.50	85.0	1643	5.51
Sd	2.27	780	3.54	2.54	790	1.49	8.94	793	2.32
# best	9	0	0	5	0	5	3	0	0
	HapTree			SCGD			ILP		
Chromosome	CPR	MEC	t(sec)	CPR	MEC	t(sec)	CPR	MEC	t(sec)
1	84.1	2305	15.43	92.5	2456	3.62	95.6	1741	173.68
2	84.5	2875	17.59	92.6	3509	4.41	95.3	2219	190.37
3	85.2	2363	15.06	91.9	2498	3.40	95.6	1788	152.09
4	83.5	2604	18.67	92.7	3754	5.47	97.1	2048	168.56
5	84.8	2171	16.95	93.9	2750	3.54	95.4	1691	147.72
6	84.6	3583	23.86	93.0	5612	8.70	95.7	2643	181.51
7	84.7	2070	13.06	93.5	2826	3.95	95.4	1590	133.36
8	84.2	1838	14.81	90.7	1692	2.18	95.6	1472	136.60
9	85.1	1479	14.90	97.1	1885	2.94	95.2	1125	105.34
10	85.7	1823	12.13	92.6	1876	2.56	95.7	1354	120.89
11	83.6	1577	11.33	93.2	2265	2.95	95.2	1206	104.74
12	84.8	1589	9.97	92.3	1612	2.03	95.4	1214	103.88
13	82.8	1405	9.55	97.0	2947	3.31	95.5	1105	93.33
14	85.4	987	7.79	91.1	904	1.36	95.3	752	65.07
15	83.6	1061	7.43	99.1	1041	1.21	94.1	809	66.52
16	85.1	1273	8.13	93.0	1305	1.79	95.5	920	77.81
17	84.8	1230	6.34	96.7	2123	2.61	96.1	943	47.99
18	84.1	941	7.13	90.3	933	1.16	95.2	720	71.49
19	84.6	765	5.26	97.2	1290	3.25	96.6	533	44.32
20	86.9	795	6.08	96.8	949	1.38	95.8	612	54.30
21	86.3	528	5.05	94.3	499	0.63	95.2	415	31.82
22	86.9	436	4.65	94.1	422	0.74	95.2	316	31.89
Mean	84.8	1623	11.42	93.9	2052	2.87	95.5	1237	104.69
Sd	1.03	802	5.23	2.3	1222	1.80	0.57	612	50.37
# best	0	0	0	1	0	17	5	22	0

The best results in each Chromosome and in all Chromosomes are in bolface font

Fosmid pool-based sequencing provides very long fragments and is characterized by much higher ratio of the number of SNPs to the number of reads than the standard data sets generated by high-throughput sequencing platforms. We consider the fosmid sequence data for chromosomes of HapMap NA12878 and again take the trio-phased variant calls from the GATK resource bundle [44] as the true haplotype sequences. We compare the performance of AltHap to those of H-PoP, BP, HapTree, SCGD and ILP and report the results in Table 2. As can be seen from Table 2, AltHap achieves the best CPR for most of the chromosomes (thirteen out of 22) followed by H-PoP (four). As with the 1000 Genome Project Data, ILP achieves the best MEC scores but is much slower and significantly inferior to AltHap in terms of CPR. Note that since HapTree could not finish assembling haplotype of the 6^*th*^ chromosome in 48 hours, that result is missing from the table.

**Table 2:** Performance comparison of AltHap, H-PoP, BP, HapTree, SCGD, and ILP applied to the Fosmid data set. HapTree could not finish assembling haplotype of the 6^*th*^ chromosome in 48 hours

	AltHap			H-PoP			BP		
Chromosome	CPR	MEC	t(sec)	CPR	MEC	t(sec)	CPR	MEC	t(sec)
1	95.5	9731	18.38	84.8	9845	2.13	87.6	9567	40.18
2	95.5	9589	38.89	90.4	9444	2.16	84.8	9698	42.90
3	91.7	7311	29.40	91.7	7182	1.79	84.7	7587	30.61
4	92.7	5508	26.69	92.6	5775	1.76	86.9	6288	31.10
5	92.0	6711	27.39	93.9	6910	1.95	86.3	6975	36.94
6	90.9	7213	33.68	88.5	7505	2.40	85.0	7590	41.20
7	90.7	6151	28.60	91.9	6829	1.68	85.8	6091	36.94
8	91.2	5927	23.82	90.2	6143	1.89	87.3	6282	38.87
9	91.8	5347	19.40	91.8	5719	1.76	85.1	5493	26.13
10	90.1	6044	24.07	92.4	6328	1.48	86.4	6503	27.65
11	90.8	5424	21.73	90.3	6432	1.68	85.8	5579	20.56
12	91.5	5456	24.25	91.4	5653	1.46	85.0	5706	24.19
13	90.4	3646	14.23	90.1	3708	1.54	82.7	3976	17.33
14	89.5	4156	18.64	89.1	4261	1.21	87.0	4004	14.84
15	90.0	4079	14.67	72.9	4001	1.06	82.3	4022	14.35
16	88.5	6197	26.28	71.5	6119	1.20	84.4	5112	29.51
17	89.7	4507	16.35	88.3	4911	1.22	87.6	4749	18.29
18	93.0	3080	12.68	90.8	3315	1.14	85.5	3457	13.31
19	85.7	4212	13.40	86.3	4115	0.84	83.5	3928	13.44
20	90.3	3512	13.64	90.0	4121	0.85	84.9	3814	15.97
21	92.7	1871	6.20	91.9	1974	0.68	87.2	1953	8.18
22	85.1	3654	17.24	87.8	3757	0.62	86.7	3910	14.72
mean	90.9	5424	21.35	88.6	5639	1.48	85.6	5558	25.33
Sd	2.5	1950	7.79	5.7	1934	0.50	1.5	1948	10.84
# best	13	0	0	4	0	9	0	0	0
	HapTree			SCGD			ILP		
Chromosome	CPR	MEC	t(sec)	CPR	MEC	t(sec)	CPR	MEC	t(sec)
1	91.5	9676	6501	95.1	10127	2.59	79.0	6889	80.33
2	92.3	9802	7196	94.5	9721	2.41	76.1	6700	76.60
3	90.7	7705	4847	88.6	7410	1.83	76.9	5122	79.50
4	90.8	6500	8392	87.6	5494	1.48	77.0	4072	51.49
5	90.8	7094	5670	89.6	7058	1.71	76.0	4637	54.39
6	-	-	-	90.4	7843	2.14	75.7	5248	63.37
7	91.5	6169	5589	89.4	6189	1.73	77.9	4174	46.85
8	91.2	6379	8316	87.4	5996	1.47	76.3	4301	53.57
9	91.7	5513	4465	90.0	5592	1.20	76.8	3974	42.41
10	88.9	6553	4838	92.8	6027	1.60	76.8	4508	59.25
11	90.5	5625	5183	90.1	5662	1.34	79.0	3903	45.45
12	91.3	5770	5654	90.5	5731	1.55	77.5	3907	48.76
13	89.8	4029	5367	87.6	3727	0.79	77.1	2669	32.09
14	90.6	4038	4103	92.9	4859	1.12	75.4	2814	39.61
15	90.7	4116	3357	87.8	4442	0.88	78.7	2903	33.80
16	94.2	5142	9683	95.5	6474	1.60	79.8	3844	62.44
17	93.1	4806	3003	97.1	4843	1.01	80.8	3448	42.00
18	91.9	3493	2303	88.3	3478	0.71	76.9	2337	32.27
19	92.8	3953	1984	82.5	4204	0.87	78.6	2707	33.68
20	90.1	3886	1529	94.6	3790	0.83	78.7	2783	31.78
21	92.1	1979	1410	90.7	2042	0.36	77.2	1367	16.42
22	92.4	3307	1351	90.6	3495	1.06	77.0	2422	60.62
mean	91.4	5502	4797.19	90.6	5645	1.38	77.6	3851	49.39
Sd	1.2	1998	2392.54	3.4	1977	0.56	1.39	1360	16.82
# best	4	0	0	4	0	13	0	22	0

The best results in each Chromosome and in all Chromosomes are in bolface font

### Simulated data: the diploid case

To further benchmark performance of the proposed scheme, we test it on the synthetic data from [39] often used to compare methods for haplotype assembly of diploids. These data sets emulate haplotype assembly under varied coverage, sequencing error rates and haplotype block lengths. We constrain our study to the assembly of haplotype blocks having length *m*=700 bp (the longest blocks in the data set). The results, averaged over 100 instances of the problem, are given in Table 3. As evident from this table, AltHap outperforms other algorithms for nearly all the combinations of data error rates and sequencing coverage and is also much faster than SCGD, ILP, BP and HapTree while being slightly slower than H-PoP. Note that ILP could only finish assembling haplotype of two settings with *p*_*e*_=0.1 and coverages of 5 and 8, in 48 hours. Hence, the results for other settings are missing from the table.

**Table 3:** Performance comparison of AltHap, H-PoP, BP, HapTree, SCGD, and ILP on a simulated diploid data set from [39] with haplotype block length *m*=700. ILP could only finish assembly of haplotypes for two settings in 48 hours

		AltHap			H-PoP			BP		
Error rate	Coverage	CPR	MEC	t(s)	CPR	MEC	t(s)	CPR	MEC	t(s)
0.1	5	99.6	477	0.043	99.3	402	0.012	86.7	698	1.421
0.1	8	99.9	759	0.128	99.8	780	0.035	87.2	861	4.627
0.1	10	99.9	954	0.404	99.9	903	0.109	87.3	1130	13.58
0.2	5	90.9	941	0.061	87.7	1021	0.027	81.2	953	2.671
0.2	8	98.1	1458	0.141	88.9	1532	0.098	86.1	1847	6.897
0.2	10	99.1	1836	0.394	91.5	2023	0.201	86.7	2485	10.13
0.3	5	60.7	1228	0.069	61.8	1331	0.041	53.7	1677	3.235
0.3	8	67.7	2022	0.145	65.7	2250	0.098	57.2	2469	7.982
0.3	10	75.0	2558	0.375	71.2	2979	0.217	59.6	3114	15.32
		HapTree			SCGD			ILP		
Error rate	Coverage	CPR	MEC	t(s)	CPR	MEC	t(s)	CPR	MEC	t(s)
0.1	5	88.6	491	2.13	96.6	523	0.66	98.8	467	471
0.1	8	88.4	767	3.82	99.8	772	0.84	99.7	760	2004
0.1	10	87.3	963	4.03	99.9	965	0.97	-	-	-
0.2	5	76.2	988	9.36	76.1	979	0.72	-	-	-
0.2	8	80.8	1562	6.69	91.3	1531	1.18	-	-	-
0.2	10	82.7	1943	4.20	95.4	1902	1.50	-	-	-
0.3	5	64.6	1170	10.21	57.8	1136	0.73	-	-	-
0.3	8	65.7	2021	6.17	63.7	1998	1.14	-	-	-
0.3	10	65.1	2597	5.74	67.9	2574	1.44	-	-	-

The best results in each simulation setting are in bolface font

### Simulated data: the biallelic polyploid case

The performance of AltHap in applications to haplotype assembly for polyploids was tested using simulations; in particular, we studied how AltHap’s accuracy depends on coverage and sequencing error rate. The generated data sets consist of paired-end reads with long inserts that emulate the scenario where long connected haplotype blocks need to be assembled. We simulate sampling of the entire genome using paired-end reads and generate SNPs along the genome with probability 1 in 300. In other words, the distance between pairs of adjacent SNPs follows a geometric random variable with parameter $p_{snp}=\frac {1}{300}$ (the SNP rate). To emulate a sequencing process capable of facilitating reconstruction of long haplotype blocks, we randomly generate paired-end reads of length 2×250 with average insert length of 10,000 bp and the standard deviation of 10%; sequencing errors are inserted using realistic error profiles [45] and genotyping is performed using a Bayesian approach [44]. At such read and insert lengths, the generated haplotype blocks are nearly fully connected. Each experiment is repeated 10 times. AltHap is compared with H-PoP, BP and SCGD. We also tried to run HapTree. However, HapTree could not finish the simulations for the considered block size in 48 hours.

Table 4 compares the CPR, MEC score, and running times of AltHap with those of H-PoP, BP and SCGD for biallelic triploid genomes with haplotype block lengths of *m*=1000 for several combinations of sequencing coverage and data error rates. As can be seen there, in terms of CPR AltHap outperforms all other methods in all the scenarios while in terms of MEC score it outperforms other methods in the vast majority of the scenarios. Note that unlike H-PoP’s, the complexity of AltHap scales gracefully with coverage (i.e., although H-PoP is very fast at low coverages, at the highest coverage AltHap becomes much faster than H-PoP). As can be seen in Table 6, overall CPR score (MEC score) of all algorithms decreases (increases) as the probability of error increases. This is expected – and also reflected in our theoretical results – since with higher data error rate haplotype assembly becomes more challenging. Additionally, MEC scores increases with coverage since higher coverage implies more sequencing reads. Therefore, the total number of observed SNP positions increases which in turn results in higher MEC scores.

**Table 4:** Performance comparison of AltHap, H-PoP, BP, and SCGD on a simulated biallelic triploid data set with haplotype block length *m*=1000. HapTree could not finish the simulations in 48 hours

		AltHap			H-PoP			BP			SCGD		
Err	Cov	CPR	MEC	t(s)	CPR	MEC	t(s)	CPR	MEC	t(s)	CPR	MEC	t(s)
0.002	10	**98.2**	**322**	30	71.5	3642	**14**	68.9	4210	132	69.7	11988	159
0.002	20	**95.1**	**1986**	59	73.1	7728	**41**	72.9	7762	416	51.8	35660	283
0.002	30	98.4	2412	109	70.8	12865	265	69.7	14751	1310	52.1	53248	422
0.01	10	**91.7**	**1379**	30	70.0	3786	14	68.1	4092	138	68.4	12108	161
0.01	20	**97.7**	**1597**	60	70.9	8375	**42**	68.9	8601	460	52.0	35606	295
0.01	30	98.9	3143	110	71.8	11769	266	68.1	15124	1301	52.7	53185	422
0.05	10	**97.1**	**2802**	31	70.1	3978	14	66.9	4227	135	67.5	13037	158
0.05	20	**94.9**	**8222**	59	70.3	9276	**41**	70.1	9484	460	51.7	35693	285
0.05	30	82.6	17284	110	71.3	13778	268	67.6	16876	1315	52.1	52499	431

The best results in each simulation setting are in bolface font

The results of tests conducted on simulated biallelic tetraploid genomes are summarized in Table 5, where we observe that AltHap outperforms the competing schemes in terms of both accuracy and running time. To investigate how the performance and complexity of AltHap vary with coverage and read length, in Table 6 we report its CPR, MEC, and runtimes obtained by simulating assembly of biallelic triploid haplotypes using paired end reads of length 2×250, 2×300, and 2×500 and coverage 10, 20 and 30 (block length is set to *m*=1000 and data error rate is *p*_*e*_=0.002). The results imply that AltHap’s runtime scales approximately linearly with both read length and coverage over the consider range of these two parameters. Additionally, as we see in Table 5, MEC score slightly increases with read length. The impact of read length in this matter is similar to that of sequencing coverage. longer sequencing reads provide more observed SNP positions and hence the MEC might increase, as also predicted by our theoretical results.

**Table 5:** Performance comparison of AltHap, H-PoP, BP, and SCGD on a simulated biallelic tetraploid data set with haplotype block length *m*=1000. HapTree could not finish the simulations in 48 hours

		AltHap			H-PoP			BP			SCGD		
Err	Cov	CPR	MEC	t(s)	CPR	MEC	t(s)	CPR	MEC	t(s)	CPR	MEC	t(s)
0.002	10	91.1	**1113**	43	70.7	3366	43	69.8	4568	290	67.1	14839	208
0.002	20	95.0	**2113**	87	73.4	7359	113	71.2	9434	540	51.7	41241	419
0.002	30	99.9	674	163	72.6	11693	598	71.5	12745	1496	51.8	61885	653
0.01	10	98.2	**938**	44	69.3	3511	46	66.4	6475	296	67.1	14819	213
0.01	20	99.3	**1668**	87	70.3	7882	114	66.9	10213	552	51.5	41712	414
0.01	30	95.3	6518	164	71.0	12392	597	68.4	13245	1485	51.5	61981	652
0.05	10	93.7	**3905**	44	67.7	4110	46	64.5	6869	306	65.0	15861	213
0.05	20	95.8	9645	89	69.1	9109	118	68.5	11477	623	51.9	41042	408
0.05	30	81.5	18690	165	70.0	14212	601	67.5	17681	1504	51.7	62261	643

The best results in each simulation setting are in bolface font

**Table 6:** Performance of AltHap on simulated biallelic triploid data set with haplotype block length *m*=1000, data error rate *p*_*e*_=0.002, and different read lengths

Read length	Cov	CPR	MEC	t(s)
2 × 250	10	98.2	322	30.74
2 × 250	20	95.1	1986	59.65
2 × 250	30	98.4	2412	109.73
2 × 300	10	93.0	856	34.83
2 × 300	20	97.9	1410	66.50
2 × 300	30	97.7	3216	117.62
2 × 500	10	95.5	682	39.36
2 × 500	20	92.4	2605	66.37
2 × 500	30	93.0	5869	116.69

### Simulated data: the polyallelic polyploid case

We further studied the performance of AltHap on triploid and tetraploid organisms having polyallelic sites and the results are summarized in Tables 7 and 8, respectively. Notice that none of the competing schemes are capable of handling polyallelic genomes. Evidently, AltHap is able to reconstruct underlying haplotype sequences with competitive performance at a low computational cost.

**Table 7:** Performance of AltHap on simulated polyallelic triploid data set with haplotype block length *m*=1000. H-PoP, BP, HapTree, and SCGD cannot assemble polyallelic polyploid haplotypes

Error rate	Cov	CPR	MEC	t(s)
0.002	5	83.2	1377	43.05
0.002	10	93.2	897	115.13
0.002	15	93.5	1799	173.55
0.002	20	95.2	2346	232.07
0.01	5	74.7	2341	58.13
0.01	10	94.4	1269	115.41
0.01	15	90.9	3755	173.38
0.01	20	85.5	7272	235.86
0.05	5	79.9	3076	57.77
0.05	10	89.4	3925	116.33
0.05	15	93.1	6100	174.37
0.05	20	93.9	9120	236.73

**Table 8:** Performance of AltHap on simulated polyallelic tetraploid data set with haplotype block length *m*=1000. H-PoP, BP, HapTree, and SCGD cannot assemble polyallelic polyploid haplotypes

Error rate	Cov	CPR	MEC	t(s)
0.002	5	79.4	2380	109.00
0.002	10	86.5	2043	220.6
0.002	15	93.8	2148	328.49
0.002	20	96.3	2388	432.28
0.01	5	79.7	2398	113.08
0.01	10	84.1	2927	220.33
0.01	15	82.8	5787	327.10
0.01	20	99.2	2319	432.85
0.05	5	74.6	4721	113.38
0.05	10	89.0	5146	211.43
0.05	15	92.3	7555	327.20
0.05	20	92.0	13704	435.15

The results of these extensive simulations imply that, as expected, haplotype assembly becomes more challenging as the number of haplotype sequences (i.e., the ploidy) increases. Nevertheless, in all the conducted studies, AltHap consistently reconstructs haplotype sequences accurately and with practically feasible computational cost. In addition, the results of Tables 4 and 5 demonstrate that the computational time of AltHap grows significantly slower with coverage than the computational time of the competing schemes. In particular, for high coverages that are characteristic of high-throughput sequencing technologies, AltHap is the most efficient among the considered algorithm.

### CPR lower bound

**Fig. 3:**
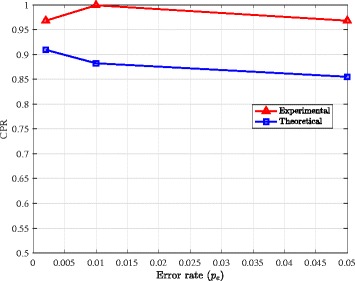
Comparison of the theoretical and experimental results. Comparison of the theoretical bound on CPR with the experimental results when *C*_seq_=15 obtained by applying AltHap to the problem of reconstructing biallelic triploid haplotypes (synthetic data)

**Fig. 4:**
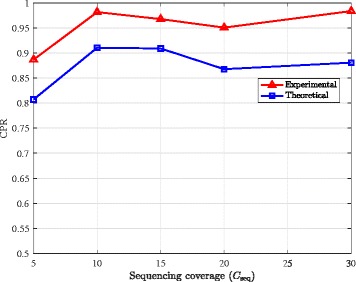
Comparison of the theoretical and experimental results. Comparison of the theoretical bound on CPR with the experimental results when *p*_*e*_=0.002 obtained by applying AltHap to the problem of reconstructing biallelic triploid haplotypes (synthetic data)

Finally, we use the results obtained by running AltHap on simulated biallelic triploid data (i.e., the results summarized in Table 4) to examine tightness of the theoretical bounds on the CPR stated in Theorem 2. In particular, theoretical bounds on CPR are compared to the CPRs empirically computed for various combinations of coverage and data error rates (averaged over 10 independent problem instances). In Fig. 3, the theoretical bound and experimental CPR results are shown as functions of the data error rate for coverage 15. We observe that the bound is reasonably close to the experimental results over the considered range of data error rates. In Fig. 4, the theoretical bound and experimental CPR results are plotted against sequencing coverage for the data error rate *p*_*e*_=0.002. This figure, too, implies that the theoretical CPR bound is relatively close to the experimental results. Notice that as Fig. 3 shows, the CPR score as well as the lower bound derived in Theorem 2 decrease when the sequencing error increases. On the other hand, Fig. 4 depicts that higher coverage improves AltHap’s CPR score, which again is reflected in our theoretical results.

## Conclusion

In this paper, we developed a novel haplotype assembly framework for both diploid and polyploid organisms that relies on sparse tensor decomposition. The proposed algorithm, referred to as AltHap, exploits structural properties of the problem to efficiently find tensor factors and thus assemble haplotypes in an iterative fashion, alternating between two computationally tractable optimization tasks. If the algorithm starts the iterations from an appropriately selected initial point, AltHap converges to a stationary point which is with high probability in close proximity of the solution that is optimal in the MEC sense. In addition, we analyzed the performance and convergence properties of AltHap and found bounds on its achievable MEC score and the correct phasing rate. AltHap, unlike the majority of existing methods for haplotype assembly for polyploids, is capable of reconstructing haplotypes with polyallelic sites, making it useful in a number of applications involving plant genomes. Our extensive tests on real and simulated data demonstrate that AltHap compares favorably to competing methods in applications to haplotype assembly of diploids, and significantly outperforms existing techniques when applied to haplotype assembly of polyploids.

As part of the future work, it is of interest to extend the sparse tensor decomposition framework to viral quasispecies reconstruction and recovery of bacterial haplotypes from metagenomic data.

## Additional file

Additional file 1 Supplement for “Sparse Tensor Decomposition for Haplotype Assembly of Diploids and Polyploids”. Additional file 1 provides details on derivation of the proposed step size, and derivation of MEC and CPR bounds. (PDF 210 kb)

## Abbreviations

BP: Belief propagation
CPR: Correct phasing rate
FPT: Fixed parameter tractable
ILP: Integer linear programming
MCMC: Markov chain Monte Carlo
MEC: Minimum error correction
SNP: Single nucleotide polymorphisms
