# Supplementary material for: Sparse Tensor Decomposition for Haplotype Assembly of Diploids and Polyploids
Source: BMC Genomics. 2018 Mar 21;19(Suppl 4):191. doi: 10.1186/s12864-018-4551-y (PMC5872563; doi:10.1186/s12864-018-4551-y)
Supplement: Supplementary file 1 — Supplement for “Sparse Tensor Decomposition for Haplotype Assembly of Diploids and Polyploids”. Additional file 1 provides details on derivation of the proposed step size, and derivation of MEC and CPR bounds. (PDF 210 kb) [file 12864_2018_4551_MOESM1_ESM.pdf]

Supplement for Sparse Tensor Decomposition for Haplotype Assembly of Diploids and Polyploids

Abolfazl Hashemi[1*], Banghua Zhu[2] and Haris Vikalo[1]

Derivation of the proposed step size

The proof relies on the results of Theorem 1 in [1]. For the proposed algorithm to converge, it must hold that

$$f(\mathbf{U}_{t+1}, \overline{\mathbf{V}}_{t+1}) \leq f(\mathbf{U}_t, \overline{\mathbf{V}}_t), \quad (1)$$

$\forall t$. First, by noting (9) it holds that $f(\mathbf{U}_{t+1}, \overline{\mathbf{V}}_t) \leq f(\mathbf{U}_t, \overline{\mathbf{V}}_t)$. It now remains to show that $f(\mathbf{U}_{t+1}, \overline{\mathbf{V}}_{t+1}) \leq f(\mathbf{U}_{t+1}, \overline{\mathbf{V}}_t)$. First, for the sake of notations and clarity, define

$$\mathrm{Tr}_\Omega(\mathbf{A}^\top\mathbf{B}) = \mathrm{Tr}\left(\mathcal{P}_\Omega(\mathbf{A}^\top)\mathcal{P}_\Omega(\mathbf{B})\right) = \sum_{(i,j)\in\Omega} \mathbf{A}_{ij}\mathbf{B}_{ij}. \quad (2)$$

Recall that $\nabla f(\overline{\mathbf{V}}_t) = -\left(\mathcal{P}_\Omega(\overline{\mathbf{R}} - \mathbf{U}_{t+1}\overline{\mathbf{V}}_t^\top)\right)^\top \mathbf{U}_{t+1}$ and $\overline{\mathbf{V}}_{t+1} = \Pi_{\mathcal{C}}(\tilde{\mathbf{V}}_{t+1})$ where $\tilde{\mathbf{V}}_{t+1} = \overline{\mathbf{V}}_t - \alpha\nabla f(\overline{\mathbf{V}}_t)$. Since $\Pi_{\mathcal{C}}$ is a projection onto a convex set of constraints, $f(\mathbf{U}_{t+1}, \overline{\mathbf{V}}_{t+1}) \leq f(\mathbf{U}_{t+1}, \tilde{\mathbf{V}}_{t+1})$. Thus, it remains to show $f(\mathbf{U}_{t+1}, \tilde{\mathbf{V}}_{t+1}) \leq f(\mathbf{U}_{t+1}, \overline{\mathbf{V}}_t)$. Given that,

$$\begin{aligned} &f(\mathbf{U}_{t+1}, \tilde{\mathbf{V}}_{t+1}) - f(\mathbf{U}_{t+1}, \overline{\mathbf{V}}_t) \\ &= \frac{1}{2}\|\mathcal{P}_\Omega(\overline{\mathbf{R}} - \mathbf{U}_{t+1}\overline{\mathbf{V}}_t^\top) + \alpha\mathcal{P}_\Omega(\mathbf{U}_{t+1}\nabla f(\overline{\mathbf{V}}_t)^\top)\|_F^2 \\ &\quad - \frac{1}{2}\|\mathcal{P}_\Omega(\overline{\mathbf{R}} - \mathbf{U}_{t+1}\overline{\mathbf{V}}_t^\top)\|_F^2 \\ &= \alpha\mathrm{Tr}(\mathcal{P}_\Omega(\overline{\mathbf{R}} - \mathbf{U}_{t+1}\overline{\mathbf{V}}_t^\top)^\top\mathcal{P}_\Omega(\mathbf{U}_{t+1}\nabla f(\overline{\mathbf{V}}_t)^\top)) \\ &\quad + \frac{\alpha^2}{2}\|\mathcal{P}_\Omega(\mathbf{U}_{t+1}\nabla f(\overline{\mathbf{V}}_t)^\top)\|_F^2 \end{aligned} \quad (3)$$

Now, consider the first term in the last line of (19). Following straightforward linear algebra we obtain

$$\begin{aligned} &-\mathrm{Tr}\left(\mathcal{P}_\Omega(\overline{\mathbf{R}} - \mathbf{U}_{t+1}\overline{\mathbf{V}}_t^\top)^\top\mathcal{P}_\Omega(\mathbf{U}_{t+1}\nabla f(\overline{\mathbf{V}}_t)^\top)\right) \\ &= -\mathrm{Tr}_\Omega\left((\overline{\mathbf{R}} - \mathbf{U}_{t+1}\overline{\mathbf{V}}_t^\top)^\top(\mathbf{U}_{t+1}\nabla f(\overline{\mathbf{V}}_t)^\top\right) \\ &= \mathrm{Tr}_\Omega((\overline{\mathbf{R}} - \mathbf{U}_{t+1}\overline{\mathbf{V}}_t^\top)^\top\mathbf{U}_{t+1}\mathbf{U}_{t+1}^\top\mathcal{P}_\Omega(\overline{\mathbf{R}} - \mathbf{U}_{t+1}\overline{\mathbf{V}}_t^\top)) \\ &= \mathrm{Tr}_\Omega(\mathcal{P}_\Omega(\overline{\mathbf{R}} - \mathbf{U}_{t+1}\overline{\mathbf{V}}_t^\top)^\top\mathbf{U}_{t+1}\mathbf{U}_{t+1}^\top\mathcal{P}_\Omega(\overline{\mathbf{R}} - \mathbf{U}_{t+1}\overline{\mathbf{V}}_t^\top)) \\ &= \|\mathbf{U}_{t+1}^\top\mathcal{P}_\Omega(\overline{\mathbf{R}} - \mathbf{U}_{t+1}\overline{\mathbf{V}}_t^\top)\|_F^2 = \|\nabla f(\overline{\mathbf{V}}_t)^\top\|_F^2. \end{aligned} \quad (4)$$

*Correspondence: abolfazl@utexas.edu
[1]Department of ECE, University of Texas at Austin, Austin, Texas, USA
Full list of author information is available at the end of the article

Therefore,

$$\begin{aligned} &f(\mathbf{U}_{t+1}, \tilde{\mathbf{V}}_{t+1}) - f(\mathbf{U}_{t+1}, \overline{\mathbf{V}}_t) \\ &= \frac{\alpha^2}{2}\|\mathcal{P}_\Omega(\mathbf{U}_{t+1}\nabla f(\overline{\mathbf{V}}_t)^\top)\|_F^2 - \alpha\|\nabla f(\overline{\mathbf{V}}_t)^\top\|_F^2 \\ &= (\frac{C^2}{2} - C)\frac{\|\nabla f(\overline{\mathbf{V}}_t)^\top\|_F^4}{\|\mathcal{P}_\Omega(\mathbf{U}_{t+1}\nabla f(\overline{\mathbf{V}}_t)^\top)\|_F^2} \end{aligned} \quad (5)$$

where the last equality follows according to the step size in (11). Clearly if $C \in (0,2)$ it must hold that $f(\mathbf{U}_{t+1}, \tilde{\mathbf{V}}_{t+1}) \leq f(\mathbf{U}_{t+1}, \overline{\mathbf{V}}_t)$, which in turn implies convergence.

Derivation of the MEC and CPR bounds

Recall that under conditions of Theorem 4.1, with probability $1 - \frac{1}{m^3}$ it holds that $\|\overline{\mathbf{M}} - \mathbf{U}^*\overline{\mathbf{V}}^{*\top}\|_F^2 \leq \frac{C_1\kappa^4 p_e km}{2C_{\mathrm{snp}}}$. Once the stationary point $\overline{\mathbf{M}}^* = \mathbf{U}^*\overline{\mathbf{V}}^{*\top}$ is found, Althap performs a decoding (rounding) step in order to obtain the binary solution $\hat{\mathbf{M}} = \mathbf{U}^*\hat{\mathbf{V}}^\top$. In this rounding procedure AltHap first normalizes all unfolded fibers such that sum of entries of each fiber equals 1. Then AltHap sets the largest entry of each unfolded fiber to 1 and the remaining three entries to 0. Eventually, AltHap reshapes the solution $\hat{\mathbf{M}}$ to the tensor $\underline{\hat{\mathbf{M}}}$. Note that this normalization is not required and we only consider it for the analysis purposes. Therefore, it is required to establish a bound on $\|\overline{\mathbf{M}} - \hat{\mathbf{M}}\|_F^2$. Let $\mathcal{L}$ be the set of mismatching fibers, i.e., $\forall f \in \mathcal{L}$, $\overline{\mathbf{M}}_f \neq \hat{\mathbf{M}}_f$. It is straightforward to see that $|\mathcal{L}| = \frac{1}{2}\|\overline{\mathbf{M}} - \hat{\mathbf{M}}\|_F^2$. First, notice that $\forall f \in \mathcal{L}^c$, $\|\overline{\mathbf{M}}_f - \overline{\mathbf{M}}^*{}_f\|_2^2 \geq \|\overline{\mathbf{M}}_f - \hat{\mathbf{M}}_f\|_2^2 = 0$. In addition, consider a fiber $\forall f \in \mathcal{L}$. The minimum value of $\|\overline{\mathbf{M}}_f - \overline{\mathbf{M}}^*{}_f\|_2^2$ occurs when two entries in f are both equal to 0.5 and the remaining two entries are both 0. Hence, it becomes clear that $\|\overline{\mathbf{M}}_f - \overline{\mathbf{M}}^*{}_f\|_2^2 \geq 0.5$. Thus, with probability $1 - \frac{1}{m^3}$ it holds that

$$\frac{1}{4}\|\overline{\mathbf{M}} - \hat{\mathbf{M}}\|_F^2 \leq \|\overline{\mathbf{M}} - \mathbf{U}^*\overline{\mathbf{V}}^{*\top}\|_F^2 \leq \frac{C_1\kappa^4 p_e km}{2C_{\mathrm{snp}}} \quad (6)$$

which is the desired relation. We now establish the MEC bound. Using the linearity of expectation and the above

discussion, we obtain

$$\begin{aligned}
&\frac{1}{2}\,\mathbb{E}\{\|\mathcal{P}_\Omega(\overline{\mathbf{R}}-\hat{\mathbf{M}})\|_F^2\} \\
&= \frac{1}{2}\,\mathbb{E}\{\|\mathcal{P}_\Omega(\overline{\mathbf{R}}-\overline{\mathbf{M}})+\mathcal{P}_\Omega(\overline{\mathbf{M}}-\hat{\mathbf{M}})\|_F^2\} \\
&\leq \mathbb{E}\{\|\mathcal{P}_\Omega(\overline{\mathbf{R}}-\overline{\mathbf{M}})\|_F^2\}+\mathbb{E}\{\|\mathcal{P}_\Omega(\overline{\mathbf{M}}-\hat{\mathbf{M}})\|_F^2\} \\
&= \mathbb{E}\{\|\mathcal{P}_\Omega(\overline{\mathbf{N}})\|_F^2\}+\mathbb{E}\{\|\mathcal{P}_\Omega(\overline{\mathbf{M}}-\hat{\mathbf{M}})\|_F^2\} \\
&= \mathbb{E}\{\|\mathcal{P}_\Omega(\overline{\mathbf{N}})\|_F^2\}+p\|\overline{\mathbf{M}}-\hat{\mathbf{M}}\|_F^2 \\
&\leq \mathbb{E}\{\|\mathcal{P}_\Omega(\overline{\mathbf{N}})\|_F^2\}+2C_1\kappa^4 p_e k \\
&= 2p_e m C_{\text{seq}}+2C_1\kappa^4 p_e k.
\end{aligned} \tag{7}$$

Thus, $\mathbb{E}\{\text{MEC}\} \leq 2p_e(C_{\text{seq}}m+\kappa^4 C_1 k)$. We now establish the CPR bound. The following is an equivalent definition of CPR computed using unfolded tensors of the true and the reconstructed haplotype sequences,

$$\text{CPR} = 1-\frac{1}{2mk}\min_{\mathcal{M}}\|\overline{\mathbf{V}}-\mathcal{M}(\hat{\mathbf{V}})\|_F^2, \tag{8}$$

where $\mathcal{M}$ is a one-to-one mapping from the corresponding entries of the lateral slices of $\underline{\hat{\mathbf{V}}}$ to those of $\underline{\hat{\mathbf{V}}}$. Assuming that sequencing reads uniformly sample haplotype sequences, on average, the mismatches between $\overline{\mathbf{V}}$ and $\hat{\mathbf{V}}$ contribute equally to the number of mismatches between $\overline{\mathbf{M}}$ and $\hat{\mathbf{M}}$. That is, $\frac{1}{2}\,\mathbb{E}\{\|\overline{\mathbf{M}}-\hat{\mathbf{M}}\|_F^2\} = \frac{n}{2k}\,\mathbb{E}\{\|\overline{\mathbf{V}}-\hat{\mathbf{V}}\|_F^2\}$. Therefore,

$$\begin{aligned}
\mathbb{E}\{\min_{\mathcal{M}}\|\overline{\mathbf{V}}-\mathcal{M}(\hat{\mathbf{V}})\|_F^2\} &\leq \mathbb{E}\{\|\overline{\mathbf{V}}-\hat{\mathbf{V}}\|_F^2\} \\
&= \frac{k}{n}\,\mathbb{E}\{\|\overline{\mathbf{M}}-\hat{\mathbf{M}}\|_F^2\} \\
&\leq \frac{2C_1\kappa^4 p_e k^2 m}{nC_{\text{snp}}}.
\end{aligned} \tag{9}$$

Thus, $\mathbb{E}\{\text{CPR}\} \geq 1-\frac{C_1\kappa^4 p_e k}{nC_{\text{snp}}}$ which is the desired bound.

Author details

[1]Department of ECE, University of Texas at Austin, Austin, Texas, USA. [2]EE Department, Tsinghua University, Beijing, China.

References

1. Cai, C., Sanghavi, S., Vikalo, H.: Structured low-rank matrix factorization for haplotype assembly. IEEE J. Selected Topics in Signal Proc. **10**(4), 647–657 (2016)
